# Common Data Elements Reported in Mechanical Thrombectomy for Acute Ischemic Stroke: A Systematic Review of Active Clinical Trials

**DOI:** 10.3390/brainsci12121679

**Published:** 2022-12-07

**Authors:** Sherief Ghozy, Nicole Hardy, Daniel J. Sutphin, Kevin M. Kallmes, Ramanathan Kadirvel, David F. Kallmes

**Affiliations:** 1 Department of Radiology, Mayo Clinic, Rochester, MN 55902, USA; 2 Nested Knowledge, St. Paul, MN 55117, USA; 3 Baruch College, The City University of New York, New York, NY 10010, USA; 4 Department of Neurologic Surgery, Mayo Clinic, Rochester, MN 55902, USA

**Keywords:** stroke, thrombectomy, clinical Trial, common data elements

## Abstract

Background: New trials are planned regularly to provide the highest quality of evidence and invade new occlusion territories, which requires a pre-defined reporting strategy with consistent, common data elements for more straightforward collective evidence synthesis. We sought to review all active endovascular thrombectomy trials to investigate their patient selection criteria, intervention description, and reported outcomes. Methods: A literature search was systematically conducted on clinicaltrials.gov for active trials and all intervention, inclusion criteria, and outcomes reported were extracted. A qualitative synthesis of the frequency of study design types and data elements are graphically and narratively presented. Results: A total of 32 studies were tagged and included in the final qualitative analysis. The inclusion criteria were highly variable, including different cut-offs for the last well-known baseline National Institutes of Health Stroke Scale, Alberta Stroke Program Early CT Score, and modified Rankin scale (mRS). Half of the studies (16/32) mentioned “thrombectomy” without defining which technique or device was used, and the final thrombolysis in cerebral infarction scale was provided in 19 (59.4%) studies. Heterogeneity was also present among the studies reporting a first-pass effect, both in how studies defined the outcome and in used ranges for mRS. Mortality and intracerebral hemorrhage (ICH) were more homogenous in their presentation and follow-up. Conclusions: There is a great degree of heterogeneity in the active thrombectomy trials concerning inclusion criteria, interventions used, and how outcomes are being reported.

## 1. Introduction

Globally, stroke is the second-leading cause of death and the third-leading cause of combined death and disabilities. In 2019, there were 12.2 million new cases and 6.55 million deaths from stroke worldwide [1]. Ischemic stroke formed 62.4% of new stroke cases in 2019, with 7.63 million cases, 3.29 million deaths, and 63.48 million disability-adjusted life-years [1]. From 1990 to 2019, the ischemic stroke incidence has increased by 88%, and deaths have increased by 61% [1].

Over the past years, several randomized clinical trials have assessed and validated the efficacy and safety of endovascular thrombectomy (EVT) in acute stroke patients, mainly the anterior circulation occlusion [2,3,4,5]. As a result, this evolving treatment modality is now considered the standard of care for acute ischemic stroke patients (AIS), with a concrete level 1 class A level of evidence [6,7]. Moreover, BAOCHE and ATTENTION trials have provided emerging evidence about the efficacy of EVT in the posterior circulation, even for late-presenting patients, with comparable outcomes to anterior circulation occlusions as presented in the 2022 European Stroke Organization Conference [8,9].

Considering the colossal burden of AIS and the continuously evolving neurointerventional field, new trials are planned regularly to provide the highest quality of evidence and invade new occlusion territories. This requires a pre-defined reporting strategy with consistent common data elements (CDEs) for more straightforward collective evidence synthesis. The current study reviewed all EVT active trials to overview their patient selection criteria, intervention description, and reported outcomes.

## 2. Methods

### 2.1. Literature Search and Study Selection

This systematic review was conducted in adherence to the Preferred Reporting Items for Systematic Reviews and Meta-Analyses (PRISMA) statement [10] and through the Nested Knowledge (NK) platform (https://nested-knowledge.com/, first accessed on 22 February 2022). In addition, a search of the literature was systematically conducted on *clinicaltrials.gov* for active trials. We used the following keywords: thrombectomy OR aspiration OR stentriever and limited the trial start dates to 2018–2022.

One reviewer (D.S.) performed all screening steps and was reviewed by two reviewers (N.H. and K.K.). First, titles and study descriptions of the retrieved trials were screened for relevance. This was followed by a further assessment of all clinicaltrials.gov data of the initially included trials for their eligibility according to the following inclusion criteria: the population was patients with acute ischemic stroke, the intervention was any type of thrombectomy (aspiration, stentriever, or a combination), the paper reports inclusion criteria related to symptom onset/last well-known, and the paper reports inclusion criteria related to baseline NIHSS (National Institutes of Health Stroke Scale) score. Trials starting before 2018 or not reporting on mechanical thrombectomy were excluded. The search and screening process details are captured in the PRISMA diagram (Supplementary Figure S1).

### 2.2. Tagging and Common Data Elements

All intervention, inclusion criteria, and reported outcomes were identified and tagged in the included studies using the AutoLit tagging feature on the platform. A tag was created for each major piece of the inclusion criteria, including time to last well-known, baseline NIHSS score, baseline mRS score, and baseline ASPECTs score. In addition, sub-tags were created under each of those criteria to identify the specific, nonequivalent criteria used in each trial. These sub-tags represent the heterogeneity of the reported inclusion criteria and outcomes across trials. Tagging was completed by two authors (D.S. and N.H.) and reviewed by a third independent author (K.K.).

### 2.3. Data Synthesis

A qualitative synthesis of the frequency of study design types and data elements was graphically presented in the form of a sunburst diagram on NK after the completion of tagging different studies [11]. Each slice represented a “tag” within the initial nest, and the platform could determine frequency based on the number of studies that had that tag.

## 3. Results

### 3.1. Semi-Automated Search Results 

Our initial search identified 40 studies. After removing duplicates and running the automated exclusion criteria, 40 studies were screened for inclusion. A total of eight studies were excluded after the screening, and 32 were tagged and included in the final qualitative analysis (Supplementary Figure S1).

### 3.2. Inclusion Criteria

**Figure 1:**
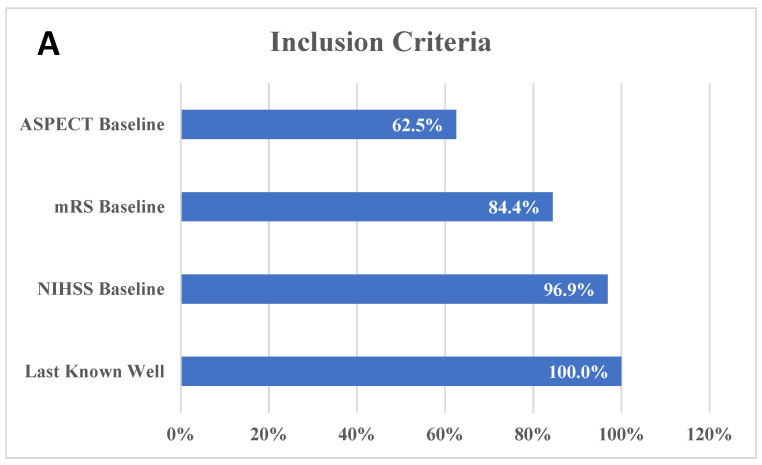

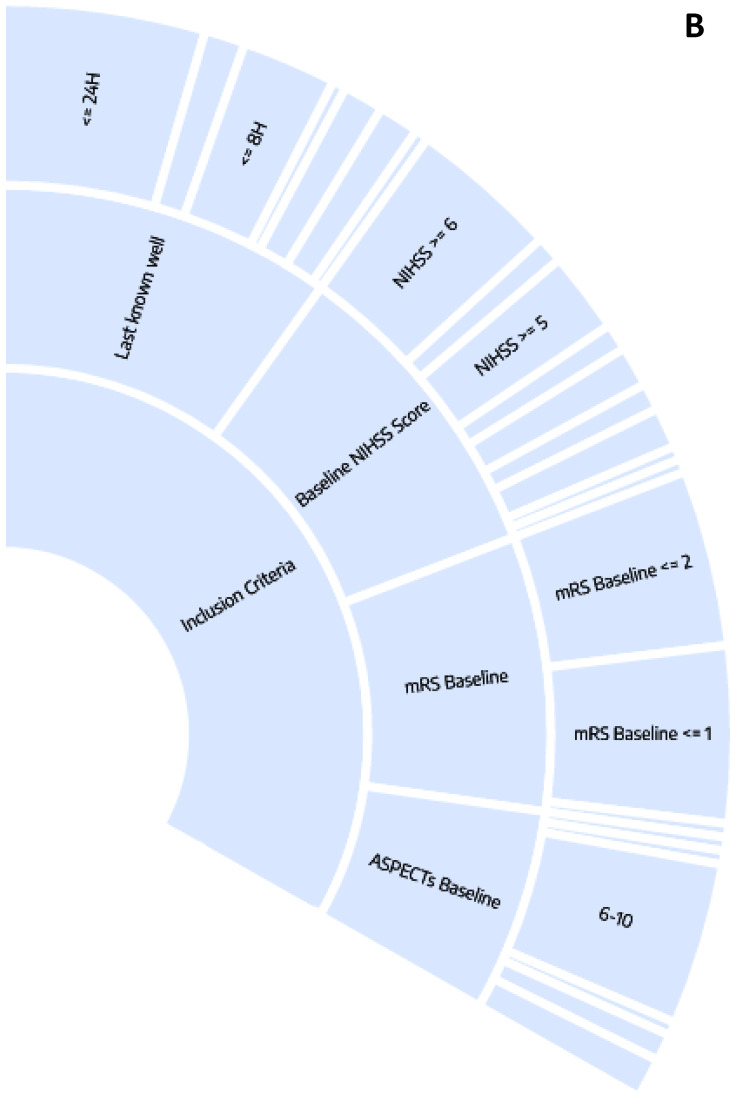
(**A**) Distribution of inclusion criteria among different studies. (**B**). Heterogeneity in different cut-offs used within inclusion criteria.

The last well-known (LWK) was the most common inclusion criteria, as all trials required it to be documented for inclusion. However, the cut-off for LWK was heterogeneous among different trials, with values ranging from ≤4.5 h to ≤24 h. The baseline National Institutes of Health Stroke Scale (NIHSS) was the second-most prevalent inclusion criteria, with 31 (96.9%) studies using this variable; nevertheless, the cut-offs used were highly variable: an NIHSS score ≥6 (34.4%) was the most commonly used, followed by ≥5 (18.8%), ≥8 (9.4%), and ≥10 (9.4%). This heterogeneity extended to the Alberta Stroke Program Early CT Score (ASPECTS), which was used in 62.5% of the studies, with variable ranges including 6–10 (37.5), 3–5 (9.4%), 5–10 (6.3%), 0–5 (3.1%), >8 (3.1%), and >4 (3.1%). On the other hand, the mRS bassline scores used were more homogenous: 27 (84.4%) studies used this criterion with mRS ≤ 1 (40.6%), while mRS ≤ 2 (40.6%) was used by almost all studies that reported baseline mRS (Figure 1).

### 3.3. Interventions

**Figure 2:**
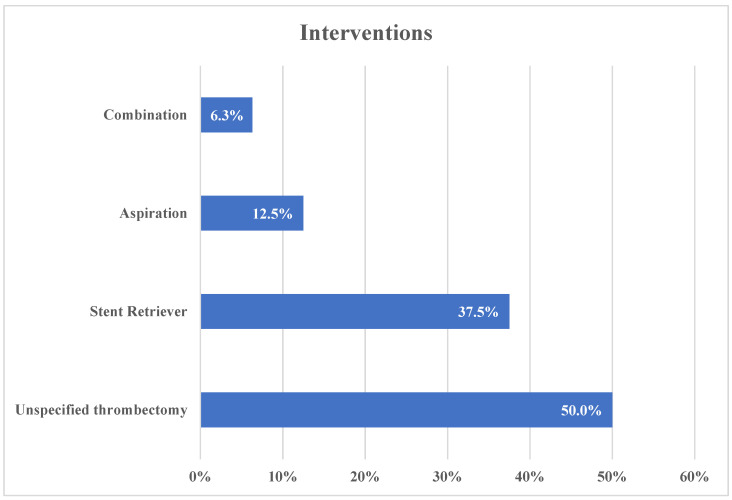
Distribution of used interventions among different studies.

Half of the studies (16/32) mentioned “thrombectomy” without defining which technique or device was used. For those who did, stent retrievers were the most commonly used technique (12/32, 37.5%), followed by aspiration (4/32, 12.5%) and a combination of both (2/32, 6.3%). Trevo/Solitaire (Stryker, Kalamazoo, MI, USA & eV3, Irvine, CA, USA, respectively) was the most commonly used stent retriever device (4/32, 12.5%), followed by Tigertriever (Rapid Medical, Yoqneam, Israel) (3/32, 9.4%), and EmboTrap II (Johnson and Johnson, New Brunswick, NJ, USA), (2/32. 6.3%). For the aspiration devices, there was an equal distribution among RapidPulse (Syntheon, Miami, FL, USA), Intracranial Thrombosis Aspiration Catheter (Sinomed Neurovita, Tianjin, China), SOFIA Flow Pulse (Microvention, Tustin, CA, USA), and Q Revascularization System (MIVI, Eden Prairie, MN, USA), with one study each. The two studies reporting combination thrombectomy used DAISe (MIVI, Eden Prairie, MN, USA) (Figure 2).

### 3.4. Reported Outcomes

**Figure 3:**
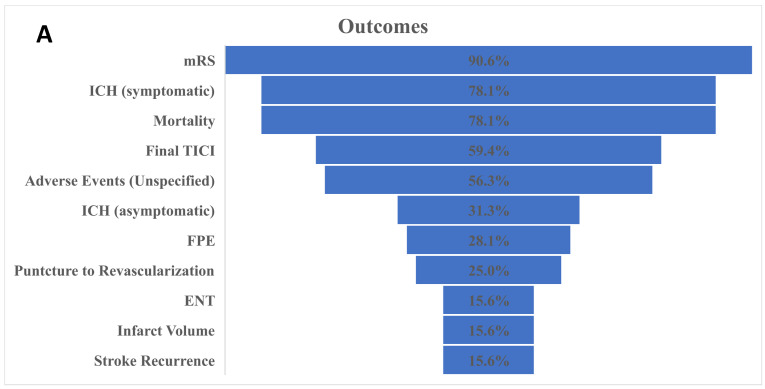

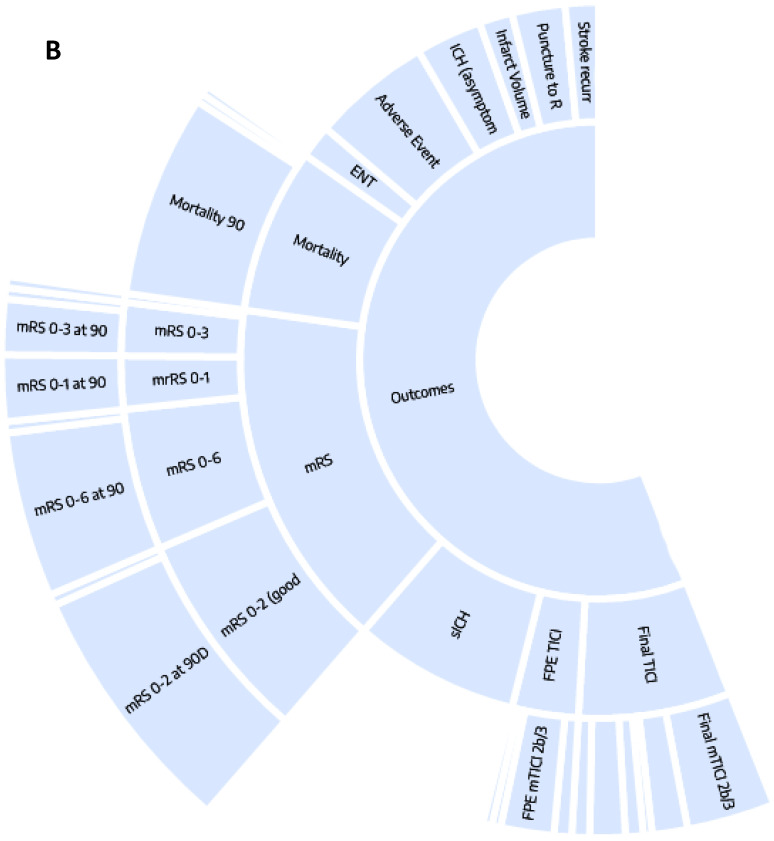
(**A**) Distribution of reported outcomes among different studies. mRS: the modified Rankin Scale; ICH: intracerebral hemorrhage; TICI: the Thrombolysis in Cerebral Infarction scale; FPE: first-pass effect; ENT: emboli to new territories. (**B**) Heterogeneity in the reported outcomes.

Recanalization rates, as reported by the final thrombolysis in cerebral infarction (TICI) scale and first-pass effect (FPE), were reported in 20 (62.5%) studies using different versions and cut-offs. The final TICI was provided in 19 (59.4%) studies, with the largest portion (10/32, 31.3%) reporting the modified TICI (mTICI) 2b/3, followed by TICI 2b–3 (4/32, 12.5%), expanded TICI (eTICI) 2b–3 (4/32, 12.5%), mTICI 2c–3 (2/32, 6.3%), TICI 0–3 (2/32, 6.3%), and TICI 3 (1/32, 3.1%), respectively. Similar heterogeneity was present among the studies reporting FPE in how studies defined the outcome. Six studies (18.8%) defined it as mTICI 2b–3, two as mTICI 2c–3 (6.3%), one as TICI 2b–3 (3.1%), and one as eTICI 2b–3 (3.1%) (Figure 3A,B).

In the same context, the functional outcome as represented by the modified Rankin scale (mRS) was the most commonly reported (29/32, 90.6%); all 29 studies measured this at the 90-day interval, and one of them provided a 180-day follow-up. However, the data presentation was heterogeneous among different studies. Most studies (23/32, 71.9%) provided a good functional outcome as defined by (mRS 0–2) at 90 days; half of them (15/32, 46.9%) provided patients’ distribution among all scale categories from zero to six, six studies (18.8%) provided the mRS 0–1, five studies provided the mRS 0–3 (15.6%), and one study presented mRS 3–6 (Figure 3A,B). 

Mortality and intracerebral hemorrhage (ICH) were more homogenous in their presentation and follow-up. Most of the studies (25/32, 78.1%) reported mortality; of those, 23 studies (71.9%) reported it at 90 days, while 30-day and one-year mortalities were reported by one study each. The symptomatic and asymptomatic ICH were both standard and reported in 25/32 (78.1%) and 10/32 (31.3%) studies, respectively. In the same context, infarct volume, puncture to revascularization, and different complications were reported homogenously and in different capacities (Figure 3B).

### 3.5. Uncommon Data Elements

**Figure 4:**
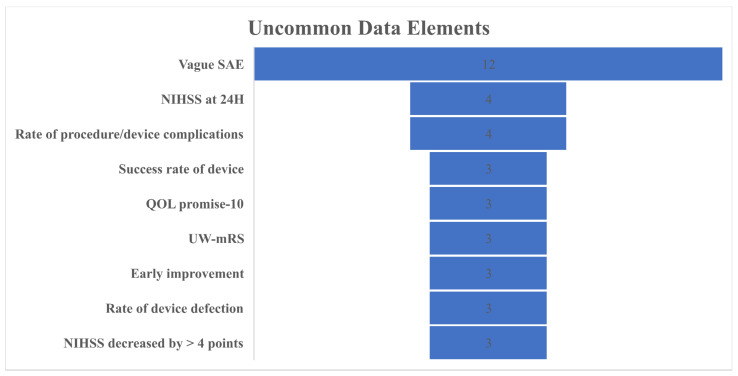
Frequency of uncommon, heterogeneously presented data elements. SAE: serious adverse events; NIHSS: National Institutes of Health Stroke Scale; QOL: quality of life; UW-mRS: utility-weighted modified Rankin Scale.

Some data elements were too heterogeneous or uncommon to be collectively presented; the most prominent ones are reported in Figure 4. There was a wide range of vaguely reported serious adverse events in 12 studies, with no specific pattern or further emphasis. For an important outcome such as NIHSS, the representation was too heterogeneous. For instance, NIHSS was reported in different studies at 24 h, 48 h, 7 days, discharge, or at 90 days. The same problem was evident in how studies defined “early improvement.” Some studies defined early improvement as NIHSS ≤ 8 or NIHSS 0–1, others as NIHSS ≤ 8 or NIHSS 0–2, and some even as NIHSS ≤ 4. Other rare representations of treatment outcomes included the utility-weighted mRS (three studies) and the quality-of-life PROMIS Global-10 (three studies). For device-related outcomes, such as complication, success rate, or defection, few studies reported them.

## 4. Discussion

The current study shows an obvious heterogeneity in thrombectomy trials in different trial stages. For instance, patient selection based on the LWK, NIHSS, ASPECT, and mRS used different cut-offs across trials, with interchangeable usage of one or more of these variables as inclusion criteria. In addition, the term “thrombectomy” was generally used with no definition of which technique or device will be used, which affects the generalizability of any results in such a mixed interventions pool. Akin to that, recanalization was reported in different capacities and definitions of what is considered successful recanalization or FPE. The same outcome-reporting disparities were present in the mRS scale, for which the follow-up duration and the definition of a good functional outcome were inconsistent. Continuing to what are mostly considered secondary outcomes, there was more homogeneity in presentation of the mortality and ICH data, with only a few differences in the last follow-up point. Notably, some outcomes were too heterogeneous and uncommon to be collectively reported, such as NIHSS at different follow-up points and intervals, serious adverse events, utility-weighted mRS, quality of life, and device-related outcomes. Our study sheds light on an impending problem that needs to be addressed appropriately.

Capturing data is one of the most expensive steps of conducting a clinical trial, and using pre-specified CDEs would improve the process of data deposition, exchange, and analysis. For that, several initiatives have tackled this matter and defined CDEs for different health conditions [12,13,14,15,16]. However, our findings show a failure in two CDE principles, with the presence of internal (reporting the same data elements in a differential manner preventing combination) and external (the lack of reporting similarities with other studies) heterogeneity. This hinders any efforts to make reliable future meta-analyses with a consolidated level of evidence, since the quality of this study design mainly depends on homogeneity in patient selection, interventions, and outcome reporting [17,18,19].

The only data element prevalent in all studies was LWK. However, it is not an indicator of homogeneity: while the studies required the LWK to be known for initial consideration, the actual recruitment was based on different cut-offs ranging from ≤4.5 to 24 h. Although several studies have shown good safety and efficacy trials for EVT up to 24 h of LWK, the functional outcomes are still variable among different intervals, limiting any future efforts to pool them together [2,3,4,5]. The encountered heterogeneity of ASPECTS categorization has been observed in large core trials, with valid concerns being raised about the inconsistency of ASPECTS grading, its ability to define “large core,” and the reliability of capturing targeted patients [20].

The included trials also showed a lack of defining the technique or device used to perform EVT. On the one hand, a recent evaluation of all meta-analyses comparing aspiration thrombectomy and stent retrievers found no concrete superiority of either technique [21]. On the other hand, devices used would affect the outcome; a network meta-analysis of randomized controlled trials concluded that using Trevo and Solitaire had higher odds of achieving good functional outcomes, while Solitaire and Aspiration had a higher safety profile. The study compared five treatment arms, including Trevo, Solitaire, Aspiration, Merci, and medical-only [22]. It should be mentioned that another meta-analysis, with observational studies inclusions, reported no differential efficacy by stent type [23].

In terms of reporting treatment outcomes, what is considered a successful recanalization was different across studies. The most recent collective evidence supports aiming for TICI 2c or TICI 3 as the new therapeutic target for the best odds of good functional outcomes [24,25,26]; however, many of the included trials seem to be sticking to some older pieces of evidence (TICI 2b is considered a success), which may be understandable due to their trial design date or designer familiarity. In the same context, most of the studies reported with mRS 0–2, which is the current standard, while others reported with mRS 0–1 and mRS 0–3. While mRS 0–1 may be justified in patients with good baseline conditions or small core infarct, mRS 0–3 is questionable. From a patient-centered view, even if many patients end up surviving with disabilities, the burden may not be acceptable. Nevertheless, this could be reasonable in trials where survival and providing the greatest benefit are prioritized, such as large core trials of posterior circulation [27,28]. Nevertheless, the ethical concerns behind trading death for a degree of disability will persist.

The current study is limited by the nature of data availability through ClinicalTrials.gov. Since the published protocols on this database may not contain all study information, some degree of reporting bias may have been introduced. We extracted all data that was available to us.

## 5. Conclusions

There is a great degree of heterogeneity in the active thrombectomy trials concerning inclusion criteria, interventions used, and how outcomes are being reported. Therefore, there is a need to adopt a standardized study design and data reporting with CDEs during the completion and publication of these studies to allow high-quality collective evidence synthesis.

## Data Availability

The data that support the findings of this study are available from the corresponding author upon reasonable request.

## Supplementary Materials

The following are available online at https://www.mdpi.com/article/10.3390/brainsci12121679/s1.
