# Supplementary figures and images for: Common Data Elements Reported in Mechanical Thrombectomy for Acute Ischemic Stroke: A Systematic Review of Active Clinical Trials
Source: Brain Sci. 2022 Dec 7;12(12):1679. doi: 10.3390/brainsci12121679 (PMC9775042; doi:10.3390/brainsci12121679)

Identification of studies via databases and registers

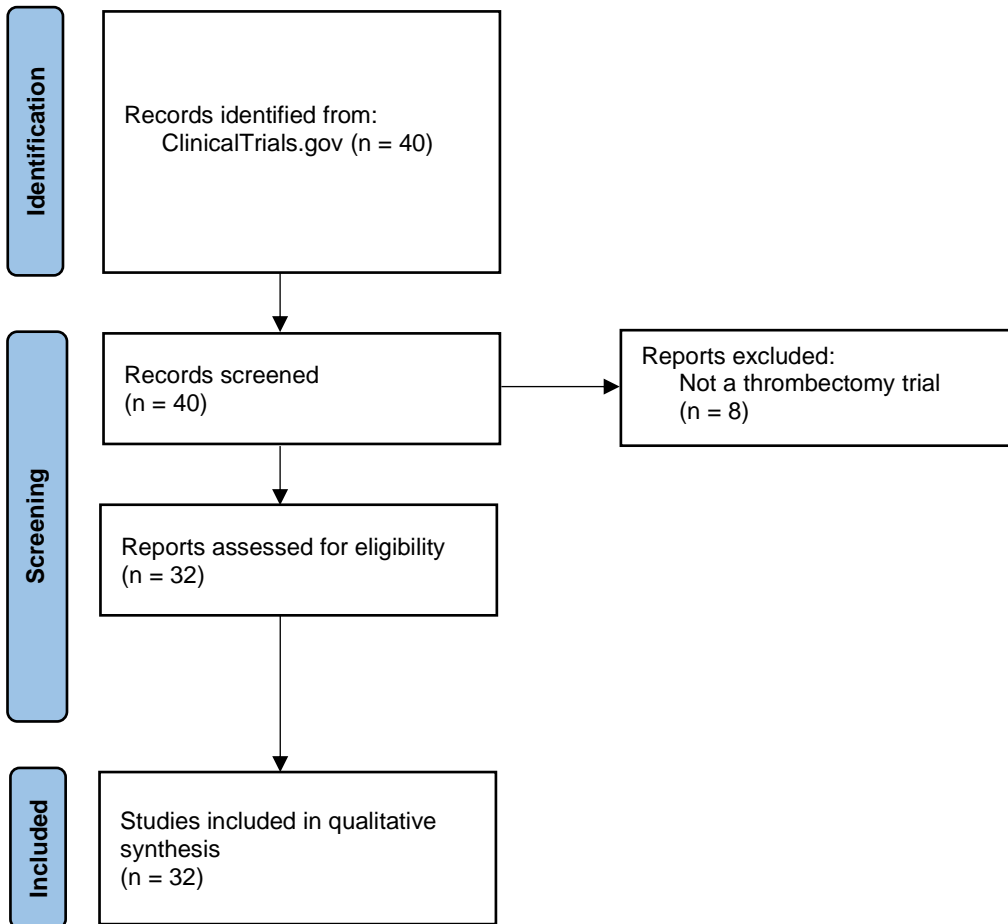

Supplement: Supplementary file 1 [file brainsci-12-01679-s001.zip › brainsci-2022841-supplementary.pdf]
